# Inflammatory biomarkers in metastatic colorectal cancer: prognostic and predictive role beyond the first line setting

**DOI:** 10.18632/oncotarget.21647

**Published:** 2017-10-04

**Authors:** Jakob Michael Riedl, Florian Posch, Florian Moik, Angelika Bezan, Joanna Szkandera, Maria Anna Smolle, Anne-Katrin Kasparek, Martin Pichler, Herbert Stöger, Michael Stotz, Armin Gerger

**Affiliations:** ^1^ Division of Clinical Oncology, Department of Medicine, Comprehensive Cancer Center Graz, Medical University of Graz, 8036 Graz, Austria; ^2^ Center for Biomarker Research in Medicine, 8010 Graz, Austria; ^3^ Department of Experimental Therapeutics, The University of Texas MD Anderson Cancer Center, Houston, TX 77054, USA

**Keywords:** biomarker, inflammation, metastatic, colorectal cancer, palliative chemotherapy

## Abstract

**Introduction:**

Inflammatory biomarkers are useful prognostic tools in cancer patients. However, the prognostic and predictive value of inflammatory biomarkers beyond the 1^st^-line setting in metastatic colorectal cancer (mCRC) is unclear.

**Results:**

In multivariate analysis 1 standard deviation increase in neutrophil-lymphocyte-ratio (NLR) was associated with an 8.5% absolute lower objective-response-rate (ORR) in 1^st^-line (p<0.0001), 3% lower ORR in 2^nd^-line (p< 0.0001), and 3% lower ORR in 3^rd^-line (p=0.24), respectively. Regarding progression free survival (PFS), an increase in the NLR was significantly associated with rising hazard-ratios (HR) over all treatment lines (HR=1.30, p= 0.021 1^st^-line); (HR=1.37, p<0.0001 2^nd^-line); (HR=1.44, p=0.042 3^rd^-line). The platelet-lymphocyte-ratio (PLR) was associated with 6-month PFS over all three treatment lines. Higher C-reactive-protein (CRP) predicted for worse PFS in the first two chemotherapy lines and in best supportive care (BSC). (HR=1.49 (p<0.0001 1^st^-line); HR=1.25 (p=0.007 2^nd^-line); HR=1.09 (95%CI 0.81–1.48, p=0.552 3^rd^-line and HR=1.43 (p= 0.002 in BSC)).

**Methods:**

Two-hundred-fifty-eight patients with mCRC undergoing palliative chemo(immuno-)therapy were retrospectively included. Primary endpoints were 6-month PFS and ORR during 1st-line, 2nd-line, and 3rd-line treatment, and 6-month overall survival during BSC.

**Conclusion:**

This study shows that inflammatory biomarkers are useful predictors of disease outcome and treatment response over several treatment lines in mCRC patients.

## INTRODUCTION

Colorectal cancer (CRC) is the third most common cancer in males and second most common in females worldwide. In developed countries the mortality rates have constantly decreased over the last years mainly due to extensive colorectal cancer screening and improved treatment options. [1] Yet, around 20 percent of patients with CRC present with synchronous metastasis at initial diagnosis and more than half of all CRC patients die from their disease. [2]

Up to date only limited data exists to predict therapy response and survival outcome in CRC patients. Since inflammation was shown to play a crucial role in the pathogenesis and promotion of cancer progression, inflammatory biomarkers have gained more attraction as potential predictive and prognostic parameters in recent years. [3, 4] A variety of routinely available blood based markers of inflammation such as hypalbuminaemia, C-reactive protein level (CRP), blood cell counts and its ratios like the neutrophil-to-lymphocyte ratio (NLR), the lymphocyte-to-monocyte ratio (LMR), or the platelet-to-lymphocyte ratio (PLR) have been investigated in different cancer entities as prognostic tools. [5–10] However, only few data exist regarding the prognosis of survival outcomes and prediction of therapy response in metastatic colorectal cancer beyond the first-line treatment setting.

The aim of this study was to examine the value of blood-based inflammatory biomarkers as prognostic and predictive markers for therapy response and disease outcome during the first three chemotherapy lines, and after start of best-supportive-care (BSC) only treatment concept in mCRC patients.

## RESULTS

### Analysis at baseline

Two-hundred-fifty-eight patients were included in this analysis (Table 1). The median age of the cohort at start of first line therapy was 66 years, and 36% were female. More than 80 % of patients had no evidence of medical comorbidity at initial diagnosis, and the median Karnofsky index was 90%. The most frequent tumor site was the rectum (n=90 (35%)), and 65 (26%) patients had right-sided tumors, which were defined as tumors located proximal to the splenic flexure. Two thirds of the patients had synchronous metastases, whereas the other third developed metastases after surgery in curative intent. Polychemotherapy regimens, which were defined as either multiagent chemotherapy or single/multiagentchemotherapy plus molecular targeted therapy were administered as 1st-line therapy in 70% of patients, as 2nd-line therapy in 62%, and as 3rd-line therapy in 56% of patients, respectively. The median NLR was 3.9 before start of first line chemotherapy. More detailed information concerning baseline demographic, tumor, treatment and laboratory variables are summarized in Table 1.

**Table 1:** Baseline characteristics of the study population

Variable	1^st^ line (n=258)		2^nd^ line (n=153)		3^rd^ line (n=72)		BSC (n=183)	
	N (%miss.)	Summary measure	N (%miss.)	Summary measure	N (%miss.)	Summary measure	n (% miss.)	Summary measure
**Demographic variables**								
Female gender	258 (0%)	92 (36%)	153(0%)	53(35%)	72(0%)	27(38%)	183(0%)	63(34%)
Age (years)	258(0%)	66 [58–73]	153(0%)	65 [59–72]	72(0%)	64 [60–71]	183(0%)	66 [59–73]
BMI (kg/m^2^)	221(14%)	24 [22–27]	134(12%)	25 [22–27]	64(11%)	24 [21–27]	0 (100%)	/
Karnofsky Index	161 (38%)	90 [80–100]	95(38%)	90 [80–90]	41(43%)	90 [80–90]	0(100%)	/
No comorbidity	256(1%)	210(82%)	151(1%)	126(83%)	70(3%)	61(87%)	182(1%)	148(81%)
Smoker or ex smoker	132(49%)	56(42%)	77(50%)	34(44%)	37(49%)	15(41%)	83(55%)	44(53%)
**Tumor variables**								
Synchronous metastases	258(0%)	172(67%)	153(0%)	104(68%)	72(0%)	48(67%)	183(0%)	121(66%)
Location of primary tumor	256(1%)	/	151(1%)	/	71(1%)	/	183(0%)	/
---Right ascending	/	43(17%)	/	22(14%)		11(15%)		33(18%)
---Right flexure	/	17(7%)	/	14(9%)		5(7%)		11(6%)
---Transverse colon	/	10(4%)	/	6(4%)		3(4%)		9(5%)
---Left flexure	/	13(5%)		7(5%)		3(4%)		13(7%)
---Left descending	/	6(2%)		5(3%)		2(3%)		5(3%)
---Sigma	/	71(28%)		37(25%)		18(25%)		45(25%)
---Rectum	/	90(35%)		56(37%)		28(39%)		62(34%)
---Multilocular	/	6(2%)		4(3%)		1(1%)		5(3%)
Kras wildtype	232(10%)	123(53%)	140(8%)	80(57%)	66(8%)	40(61%)	163(11%)	85(52%)
Nras wildtype	64(75%)	54(84%)	31(80%)	25(81%)	11(85%)	9(82%)	38(79%)	30(79%)
**Treatment variables**								
Number of chemotherapy cycles	241(7%)	8 [4–10]	141(8%)	8 [6–10]	68(6%)	8 [6–11]	/	/
Polychemotherapy	257(1%)	181(70%)	153(0%)	95(62%)	72(0%)	40(56%)	/	/
**Laboratory variables**								
Hemoglobin	232(10%)	12.4 [11.2–13.4]	119(22%)	12.7 [11.7-13.9]	59(18%)	13.1 [11.2-14.0]	164(11%)	11.4 [10.3-12.8]
Leucocyte count	194(25%)	8.8 [6.9-11.7]	120(22%)	7.1 [5.6-9.4]	59(18%)	7.6 [5.9-8.9]	165(10%)	8.5 [6.0-11.9]
Absolute neutrophil count	143(45%)	6.1 [4.4-8.7]	114(25%)	4.6 [3.4-6.3]	57(21%)	4.9 [3.5-6.0]	152(17%)	5.8 [3.9-9.2]
Absolute lymphocyte count	129(50%)	1.4 [1.1-1.9]	114(25%)	1.4 [1.0-1.7]	57(21%)	1.4 [1.0-2.0]	151(17%)	1.1 [0.8-1.7]
Absolute monocyte count	140(46%)	0.7 [0.5-0.9]	114(25%)	0.7 [0.6-0.9]	57(21%)	0.8 [0.6-1.0]	150(18%)	0.9 [0.6-1.2]
Absolute platelet count	193(25%)	312 [249–398]	120(22%)	223 [184–304]	60(17%)	252 [193–333]	164(10%)	264 [207–374]
NLR	120(53%)	3.9 [2.6-5.5]	114(25%)	3.2 [2.2-5.4]	57(21%)	3.2 [1.9-5.9]	151(17%)	5.2 [3.1-8.5]
LMR	110(57%)	1.9 [1.5-2.8]	113(26%)	1.8 [1.2-2.8]	57(21%)	1.9 [1.3-3.0]	149(19%)	1.2 [0.8-2.0]
PLR	110(57%)	212 [147–401]	114(25%)	164 [123–245]	57(21%)	171 [115–270]	150(18%)	239 [155–359]
Albumin	80(69%)	4.1 [3.6-4.4]	130(15%)	4.0 [3.7-4.2]	62(14%)	3.9 [3.5-4.1]	129(30%)	3.5 [3.0-3.8]
CRP	241(7%)	11.7 [4–34]	149(3%)	11 [4–34]	69(4%)	13.7 [5.0-48]	174(5%)	43 [12–96]
ALI^*^	170(34%)	26.9 [15.1-42.0]	88(43%)	30.9 [19.8-51.5]	46(36%)	29.5 [12.4-56.9]	N/A	N/A
Uric acid	112(57%)	5.2 [4.2-6.5]	63(59%)	5.3 [4.2-6.3]	24(67%)	5.7 [4.5-6.2]	70(62%)	5.2 [3.9-6.7]
CEA	154(40%)	17 [4–100]	135(12%)	52 [13–211]	62(14%)	67 [12–277]	130(29%)	78 [17–498]
CA19 9	143(45%)	45 [10–529]	135(12%)	83 [16–1237]	62(14%)	147 [25–1111]	127(31%)	445 [28–4406]

Distribution overall and by therapy line. The column “n (% miss.)” indicates the number of patients with observed values of the respective variable (% missing). Continuous variables are summarized as medians [25^th^ percentile (Q1) – 75^th^ percentile (Q3)], whereas categorical variables are reported as absolute frequencies and percentages. ^*^ALI = (body mass index ^*^ serum albumin) / NLR. BMI – body mass index, NLR – neutrophil to lymphocyte ratio, LMR – lymphocyte to monocyte ratio, PLR – platelet to lymphocyte ratio, CRP – C reactive protein, ALI – advanced lung cancer index.

We observed changes in the levels of the inflammatory parameters between the different treatment lines (Supplementary Table 1). The median NLR, for example, showed an 18% relative reduction from first to second line, remained at the same level after second line, but finally raised by more than 30% compared to baseline value, when entering BSC. Similar changes could be observed for the other biomarkers.

### Analysis of response patterns and their association with inflammatory biomarkers

During first-line treatment of 258 patients with chemo(immuno-)therapy, we observed 5 complete remissions (CR, 2%), 70 partial remissions (PR, 27%), 67 stable disease (SD, 26%), and 77 primary disease progressions (PD, 30%), respectively (Supplementary Figure 1). Response was not evaluable in 39 patients (NE, 15%). Response data for further lines of treatment are reported also in Supplementary Figure 1. Among the patients assessable for response, we estimated objective response rates (ORR) of 34% (95%CI: 30-41), 19% (13-26), and 17% (7-27), during 1^st^-line, 2^nd^-line, and 3^rd^-line treatment. Corresponding disease control rates (DCR, i.e. a composite of CR+PR+SD as best response) were 65% (59-71), 50% (42-59), and 37% (24-50), respectively.

In univariate analysis of absolute response rates, we observed associations between inflammatory biomarkers and ORR (Table 2). In detail and after z standardization, 1 standard deviation (SD) increase in NLR was associated with a 7% absolute lower ORR in first line (95%CI: 6-9, p<0.0001), 4% lower ORR in second line (3-5, p< 0.0001), and 2% lower ORR in third line (-1-11, p=0.68), respectively. Corresponding results for the LMR, PLR, CRP and advanced lung cancer inflammation index (ALI) are reported in the Table 2. Another strong univariate predictor of response was polychemotherapy (23% higher response rates in first line (p<0.0001), 13% higher ORR in second line (p=0.05), 20% in third line (p=0.02)). Right side location of the tumor and age were not significantly associated with ORR in all patients, but highly associated with a 26% lower 1^st^-line ORR in the subgroup of patients with KRAS-wildtype tumors (Table 2). In multivariate analysis adjusting for polychemotherapy, associations between inflammatory biomarkers and ORR prevailed (Table 2). This suggests that inflammatory biomarkers are important and independent predictive markers of response to antineoplastic chemotherapy not only in first but also in later lines of treatment.

**Table 2:** Uni and multivariate predictors of clinical response rates in first, second and third line

Variable	Δ_abs_ in 1^st^-line ORR(95%CI)	p-value	Δ_abs_ in 2^nd^-lineORR (95%CI)	p-value	Δ_abs_ in 3^rd^-line ORR(95%CI)	p-value
**Inflammatory biomarkers – Univariate analysis**						
NLR (per 1SD increase)	-7.4%(-9.1-(-5.7))	**<0.0001**	-3.6%(-4.5-(-2.7))	**<0.0001**	-2.0%(-11.9-7.8)	0.68
LMR (per 1SD increase)	5.1%(-4.2-14.5)	0.28	3.9% (-4.7-12.6)	0.38	-5.1% (-10.6-0.5)	0.07
PLR (per 1SD increase)	-2.5%(-11.7-6.7)	0.60	-4.8% (-6.8-(-2.9))	**<0.0001**	-3.3% (-5.3-(-1.2))	**0.002**
CRP (per 1SD increase)	-2.5%(-9.1-4.1)	0.45	-7.8%(-9.5-(-6.0))	**<0.0001**	4.2%(-7.0-15.5)	0.46
ALI (per 1SD increase)	8.0%(0.4-15.5)	**0.04**	10.0% (-2.0-22.0)	0.10	-7.9% (-13.0-(-2.8))	**0.002**
**Other predictors – Univariate analysis**						
Age (per 10 years increase)	-5.7%(-11.7-0.0)	0.06	-2.7% (-9.6-4.2)	0.44	-12.8%(-20.0-(-5.5))	**0.001**
Right side	-11.1%(-25.1-3.0)	0.12	2.9% (-13.0-18.8)	0.72	-15.4% (-33.2-2.4)	0.09
Right side in KRAS wildtype	-25.5%(-45.4-(-5.6))	**0.01**	7.0% (-19.2-33.2)	0.60	-22.7% (-40.2-(-5.2))	**0.01**
Polychemotherapy	22.6%(10.2-35.0)	**<0.0001**	12.7% (0.0-25.6)	**0.05**	20.5% (3.1-37.8)	**0.02**
**Inflammatory biomarkers – Multivariate analysis adjusted for polychemotherapy**						
NLR (per 1SD increase)	-8.5%(-10.5-(-6.6))	**<0.0001**	-3.0% (-4.4-(-1.6))	**<0.0001**	-3.1%(-8.3-2.0)	0.24
LMR (per 1SD increase)	4.6%(-4.6-13.8)	0.33	1.9% (-6.7-10.5)	0.67	1.1%(-8.2-10.5)	0.81
PLR (per 1SD increase)	-4.0(-12.4-4.4)	0.35	-3.0% (-7.7-(1.6))	0.20	-3.7%(-8.5-1.0)	0.13
CRP (per 1SD increase)	-3.4%(-5.3-(-1.5))	**<0.0001**	-8.4%(-8.4-(-8.3))	**<0.0001**	2.1%(-5.2-9.4)	0.58
ALI (per 1SD increase)	8.4%(1.0-15.8	**0.03)**	7.9% (-4.0-19.8)	0.20	-5.1%(-12.8-2.7)	0.20

Absolute change of ORR (objective response rate) per 1 standard deviation increase of the respective biomarker. ORR – objective response rate, CI – confidence interval, P – P value, SD – standard deviation, NLR – neutrophil to lymphocyte ratio, LMR – lymphocyte to monocyte ratio, PLR – platelet to lymphocyte ratio, CRP – C reactive protein, ALI – advanced lung cancer index.

### Univariate analysis of 6-month PFS and OS across treatment lines

Median PFS was 6.7 months in 1^st^ line, 4.2 months in 2^nd^ line and 3.2 months in 3^rd^ line therapy, respectively. Six month PFS rate was 58% (52-64), 31% (23-38) and 22% (12-33) in first, second and third line, respectively. Median OS time was 2.7 month in BSC, and 6 month OS in BSC was 32%. (Supplementary Figure 2) Associations between inflammatory biomarkers and PFS in the first three treatment lines are reported in Table 3. The Forrest plot for this analysis is shown to the end of the paragraph.

**Table 3:** Uni and multivariate predictors of clinical outcomes in first, second, third line and best supportive care

Variable	6-month PFS in 1^st^ line (HR (95%CI))	p-value	6-months PFS in 2^nd^ line (HR (95%CI))	p-value	6-months PFS in 3^rd^ line (HR (95%CI))	p-value	6-months OS in BSC (HR (95%CI))	p-value
**Inflammatory biomarkers – Univariate analysis**								
NLR (per 1SD increase)	1.21 (0.98 – 1.15)	0.083	1.39 (1.18 – 1.65)	**< 0.0001**	1.42 (1.00 – 2.02)	0.051	1.08 (0.91 – 1.28)	0.376
LMR (per 1SD increase)	0.74 (0.52 – 1.06,)	0.099	0.76 (0.58 – 1.01)	0.060	0.71 (0.48 – 1.05)	0.084	0.64 (0.46- 0.89)	0.008
PLR (per 1SD increase)	1.33 (1.02 – 1.74)	**0.036**	1.68 (1.35 – 2.10)	**< 0.0001**	1.41 (0.48 – 1.05)	**0.033**	1.16 (0.97-1.38)	0.095
CRP (per 1SD increase)	1.40 (1.16 – 1.69)	**< 0.0001**	1.25 (1.06 – 1.47)	**0.009**	1.09 (0.81 – 1.47)	0.559	1.46 (1.18 – 1.81)	**0.001**
ALI (per 1SD increase)	0.7 (0.52 – 0.95)	**< 0.024**	0.77 (0.55 – 1.08)	0.128	0.74 (0.5 – 1.08)	0.117	N/A	N/A
**Other predictors – Univariate analysis**								
Age (per 10 years increase)	1.04 (0.85 – 1.26)	0.714	1.03 (0.85 – 1.26)	0.754	1.09 (0.77 – 1.55)	0.613	0.77 (0.63 – 0.94)	**0.010**
Right side	1.31 (0.85 – 2.01)	0.218	0.84 (0.53 – 1.32)	0.440	1.32 (0.70 – 2.48)	0.390	1.46 (0.94 -2.28)	0.092
Right side in KRAS-wildtype	0.92 (0.49 – 1.74)	0.807	0.53 (0.26 – 1.08)	0.081	0.67 (0.27 – 1.65)	0.386	1.62 (0.90 – 2.92)	0.111
Polychemotherapy	0.48 (0.32 – 0.72)	**0.0001**	0.70 (0.47 – 1.04)	0.075	0.82 (0.45 – 1.48)	0.501	N/A	N/A
Metachronous metastases	1.03 (0.67 – 1.57)	0.895	0.99 (0.65 – 1.49)	0.947	1.15 (0.62 – 2.15)	0.654	0.65 (0.41-1.03)	0.067
**Inflammatory biomarkers – Multivariate analysis adjusted for polychemotherapy**	Adjusted for polychemotherapy		Adjusted for polychemotherapy		Adjusted for polychemotherapy		Adjusted for age and metachronous metastases	
NLR (per 1SD increase)	1.30 (1.04 – 1.62)	**0.021**	1.37 (1.16 – 1.62, p<0.0001)	**<0.0001**	1.44 (1.01 – 2.05)	**0.042**	1.11 (0.93 – 1.33)	0.248
LMR (per 1SD increase)	0.71 (0.49 – 1.03)	0.072	0.78 (0.59 – 1.03)	0.080	0.71 (0.48 – 1.04)	0.076	0.62 (0.44 – 0.87)	**0.006**
PLR (per 1SD increase)	1.43 (1.09 – 1.88)	0.009	1.67 (1.34 – 2.09)	**< 0.0001**	1.43 (1.04 – 1.98)	**0.029**	1.18 (0.98 – 1.43)	0.084
CRP (per 1SD increase)	1.49 (1.23 – 1.80)	**<0.0001**	1.25 (1.06 – 1.47)	**0.007**	1.09 (0.81 – 1.48)	0.552	1.43 (1.15 – 1-79)	**0.002**
ALI (per 1SD increase)	0.70 (0.51 – 0.95)	**0.022**	0.78 (0.55 – 1.09)	0.139	0.86 (0.56 – 1.33)	0.501	N/A	N/A

Hazard ratio of 6-month PFS (progression free survival) per 1 standard deviation increase of the respective biomarker. PFS – progression free survival, HR – hazard ratio, CI – confidence interval, P – P value, SD – standard deviation, NLR – neutrophil to lymphocyte ratio, LMR – lymphocyte to monocyte ratio, PLR – platelet to lymphocyte ratio, CRP – C reactive protein, ALI – advanced lung cancer index.

**Figure 1:**
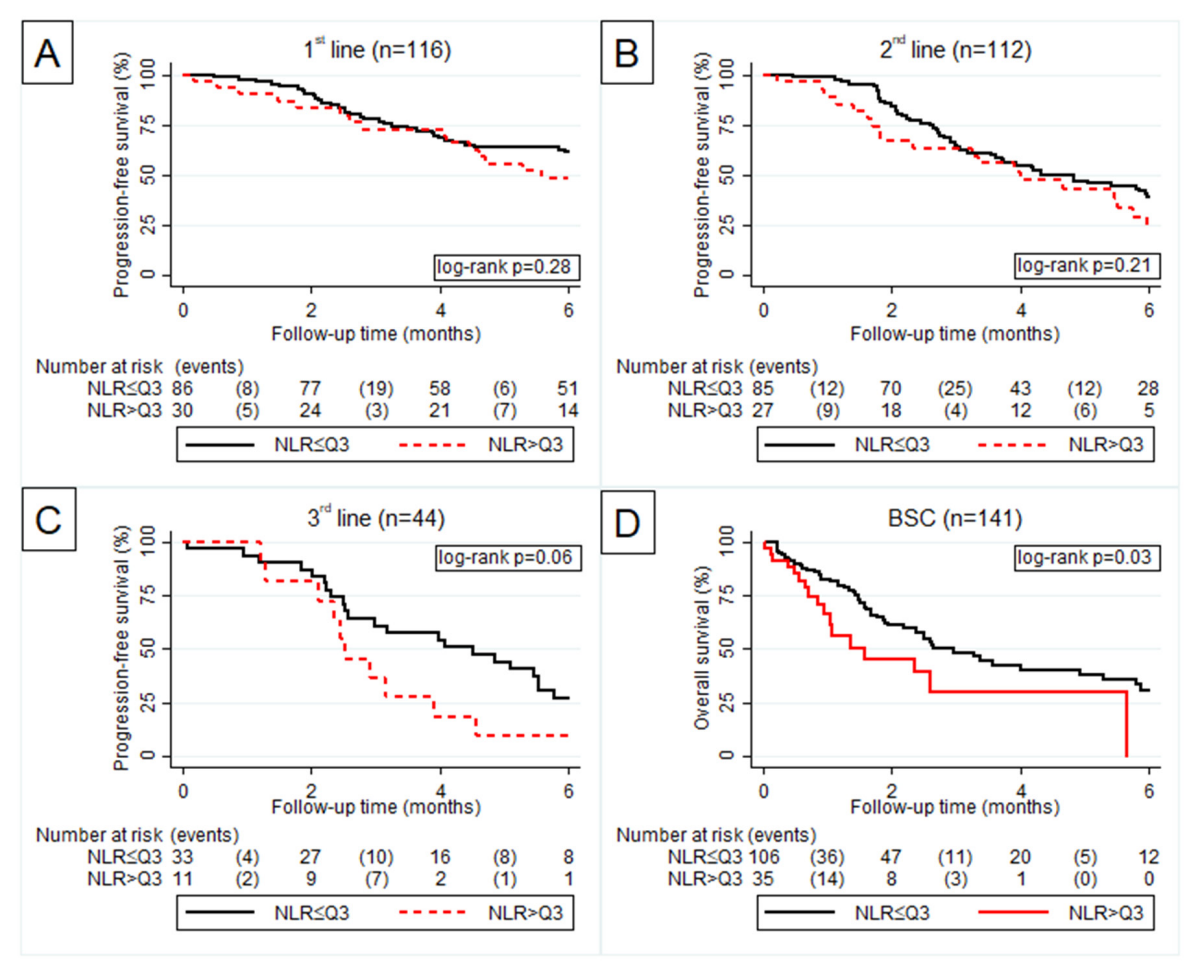
Kaplan Meier curve according to NLR > Q3 vs. NLR ≤ Q3 for progression free survival in 1^st^ **(A)**, 2^nd^ **(B)** and 3^rd^ **(C)** line of palliative chemotherapy and overall survival in best supportive care **(D)**.

In univariate Cox regression analysis, the NLR was associated with a numerically impaired 6-month PFS during first three treatment lines. However, this was only statistically significant in the 2^nd^-line setting with the numbers we had. No association between the NLR and 6-month OS could be observed in BSC (Table 3). Importantly, this was only found when using the NLR as continuous variable. When using the NLR as a dichotomized variable with empirically chosen cut-offs at the 25^th^ or 75^th^ percentile an upper quarter NLR was not significantly associated with an impaired PFS over the first three therapy lines, but with an impaired OS in BSC (Figure 1A-1D).

**Figure 2:**
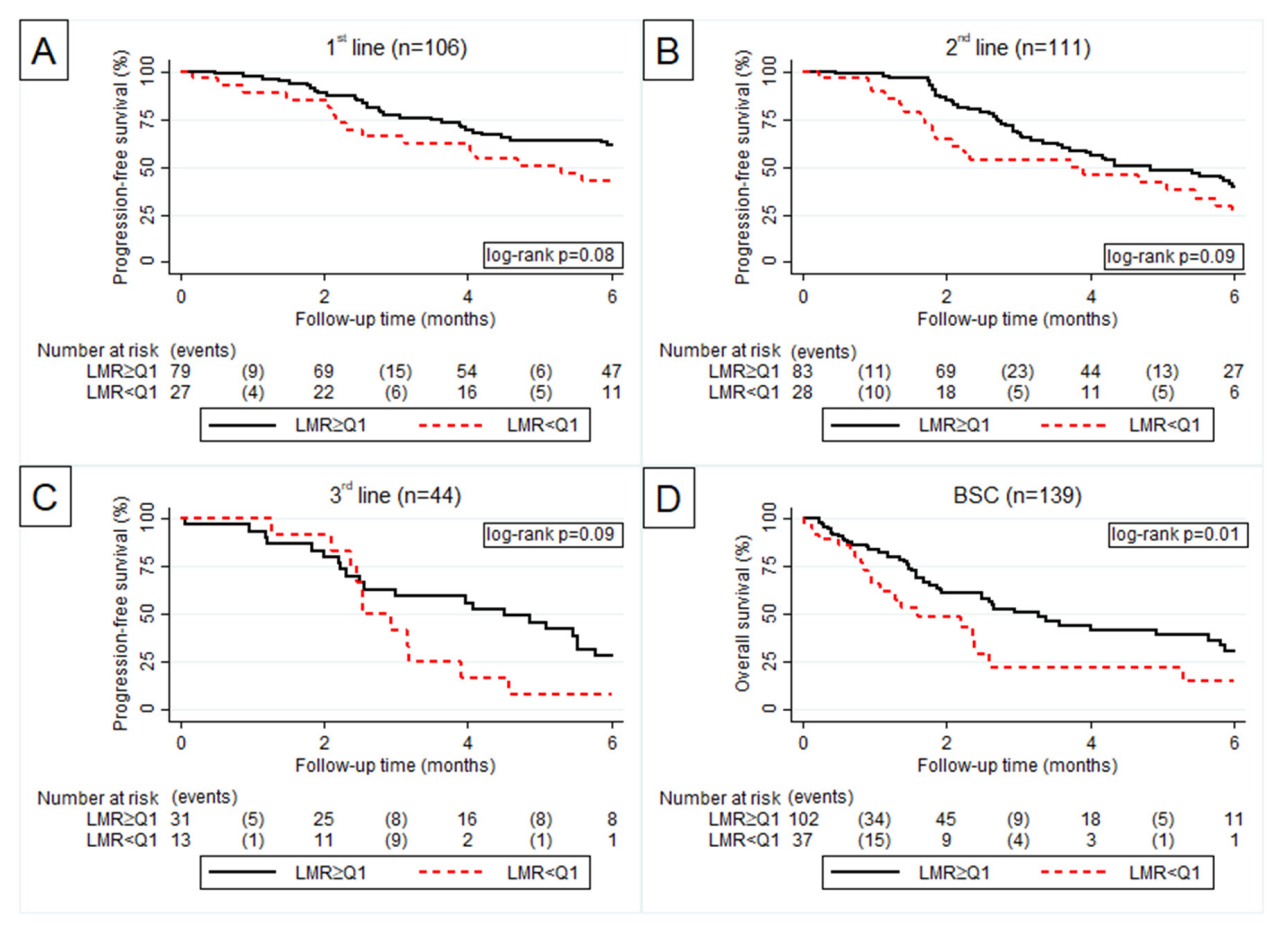
Kaplan Meier curve according to LMR > Q3 vs. LMR ≤ Q3 for progression free survival in 1^st^ **(A)**, 2^nd^ **(B)** and 3^rd^ **(C** line of palliative chemotherapy and overall survival in best supportive care **(D)**.

An elevated LMR showed a weak favourable prognostic association with PFS in all three treatment lines. (Figure 2A-2C), and was strongly associated with favourable OS prognosis in BSC (Figure 2D).

**Figure 3:**
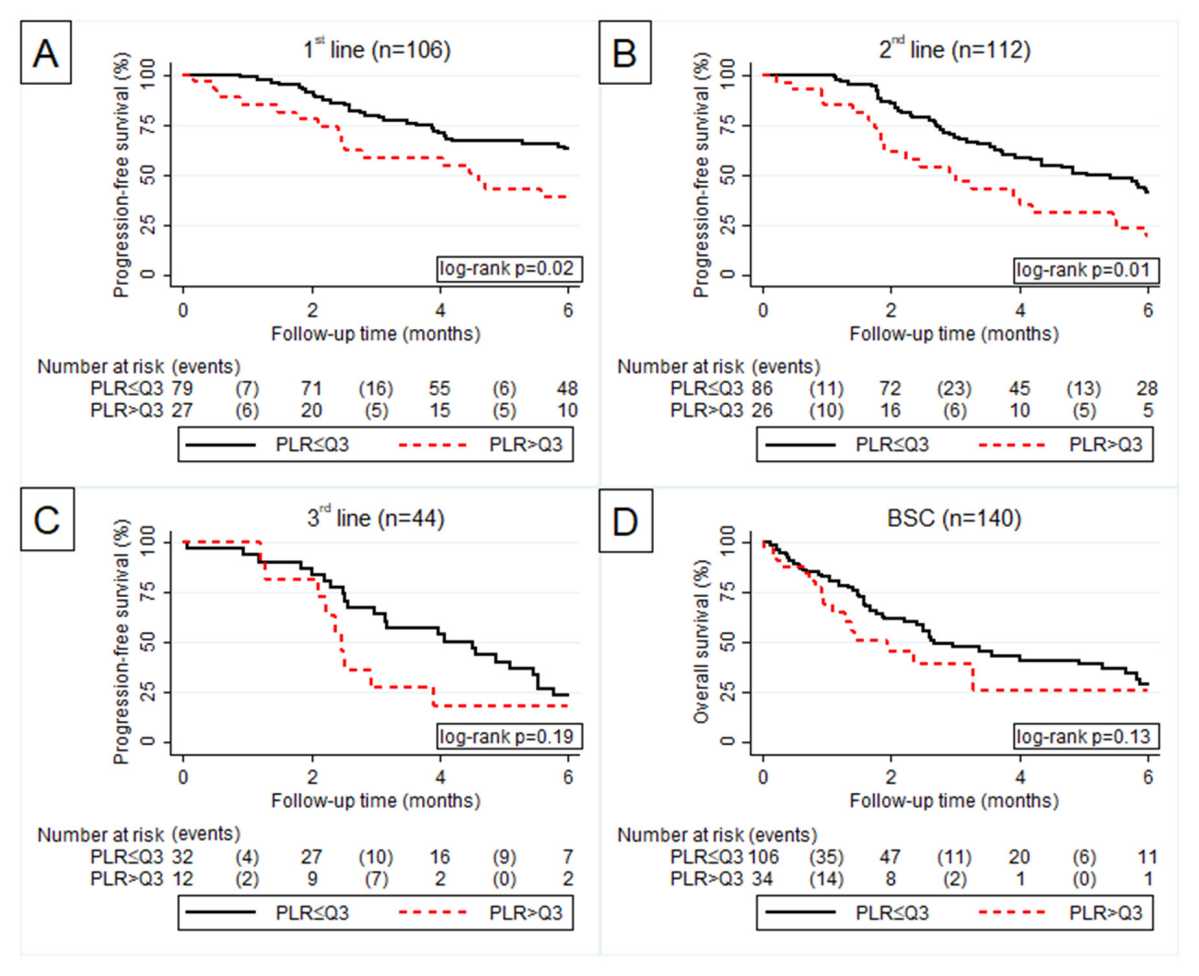
Kaplan Meier curve according to PLR > Q3 vs. PLR ≤ Q3 for progression free survival in 1^st^ **(A)**, 2^nd^ **(B)** and 3^rd^ **(C)** line of palliative chemotherapy and overall survival in best supportive care **(D)**.

An elevated PLR was a strong predictor for PFS during the first two treatment lines (Figure 3A, 3B). However this prognostic value weakened during third line and as a predictor for OS in BSC (Figure 3C, 3D).

**Figure 4:**
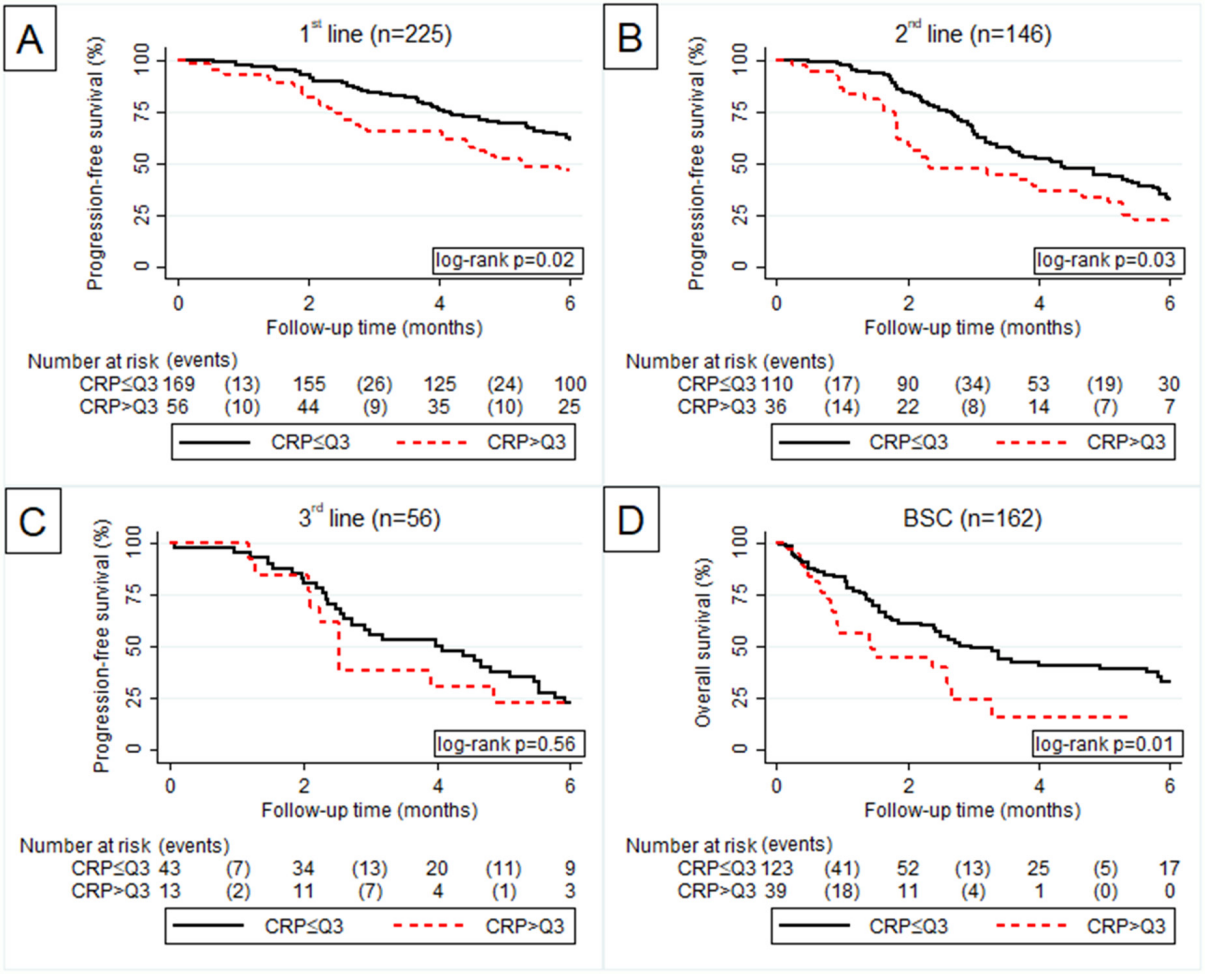
Kaplan Meier curve according to CRP > Q3 vs. CRP ≤ Q3 for progression free survival in1^st^ **(A)**, 2^nd^ **(B)** and 3^rd^ **(C)** line of palliative chemotherapy and overall survival in best supportive care **(D)**.

High CRP was strongly significantly associated with shorter PFS in first and second line and emerged as a predictor for poor OS in BSC (Figure 4A-4D). In third line no association between the CRP value and PFS could be shown (Figure 4C).

**Figure 5:**
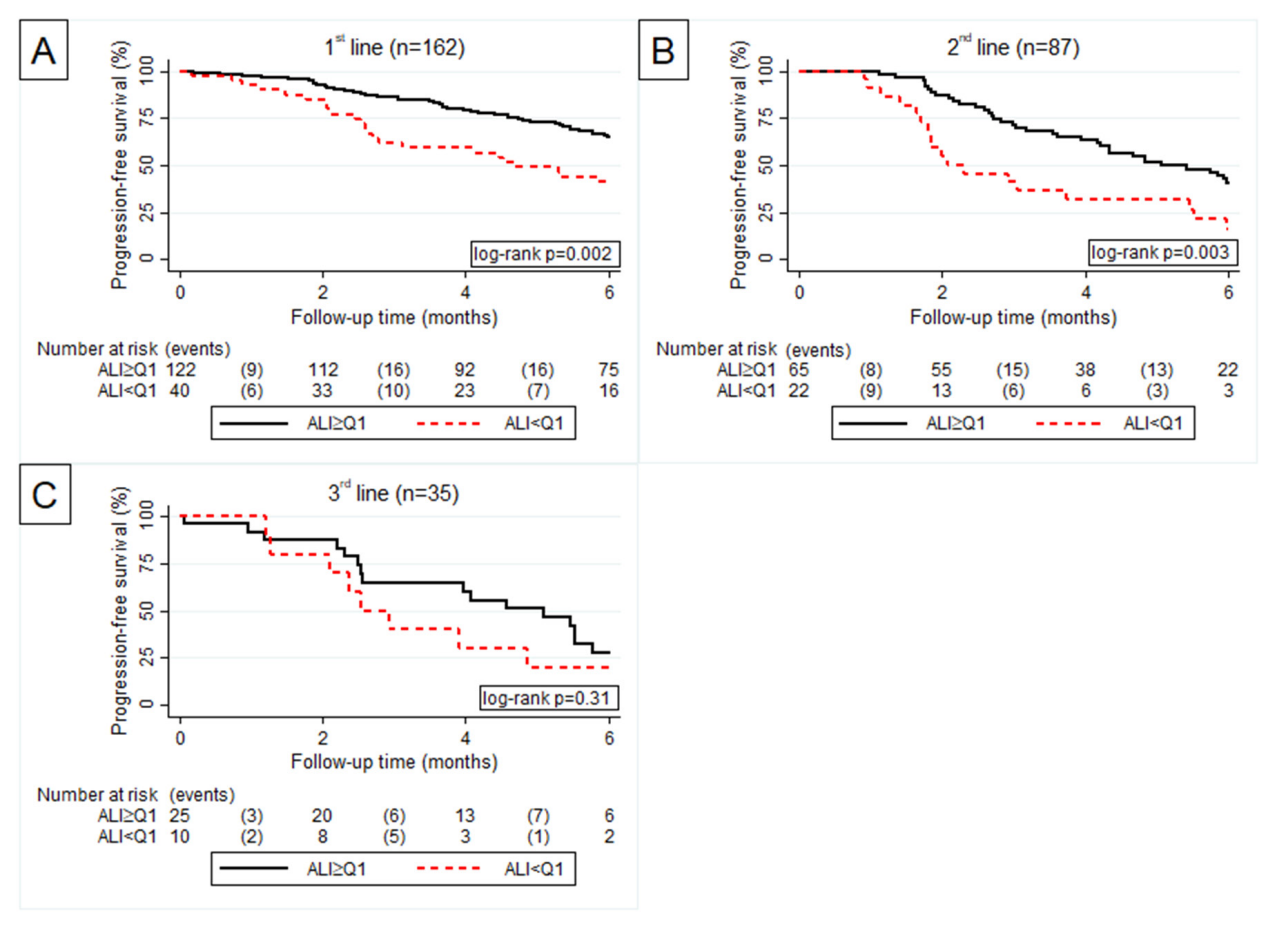
Kaplan Meier curve according to ALI > Q3 vs. ALI ≤ Q3 for progression free survival in 1^st^ **(A)**, 2^nd^ **(B)** and 3^rd^ **(C)** line of palliative chemotherapy.

**Figure 6:**
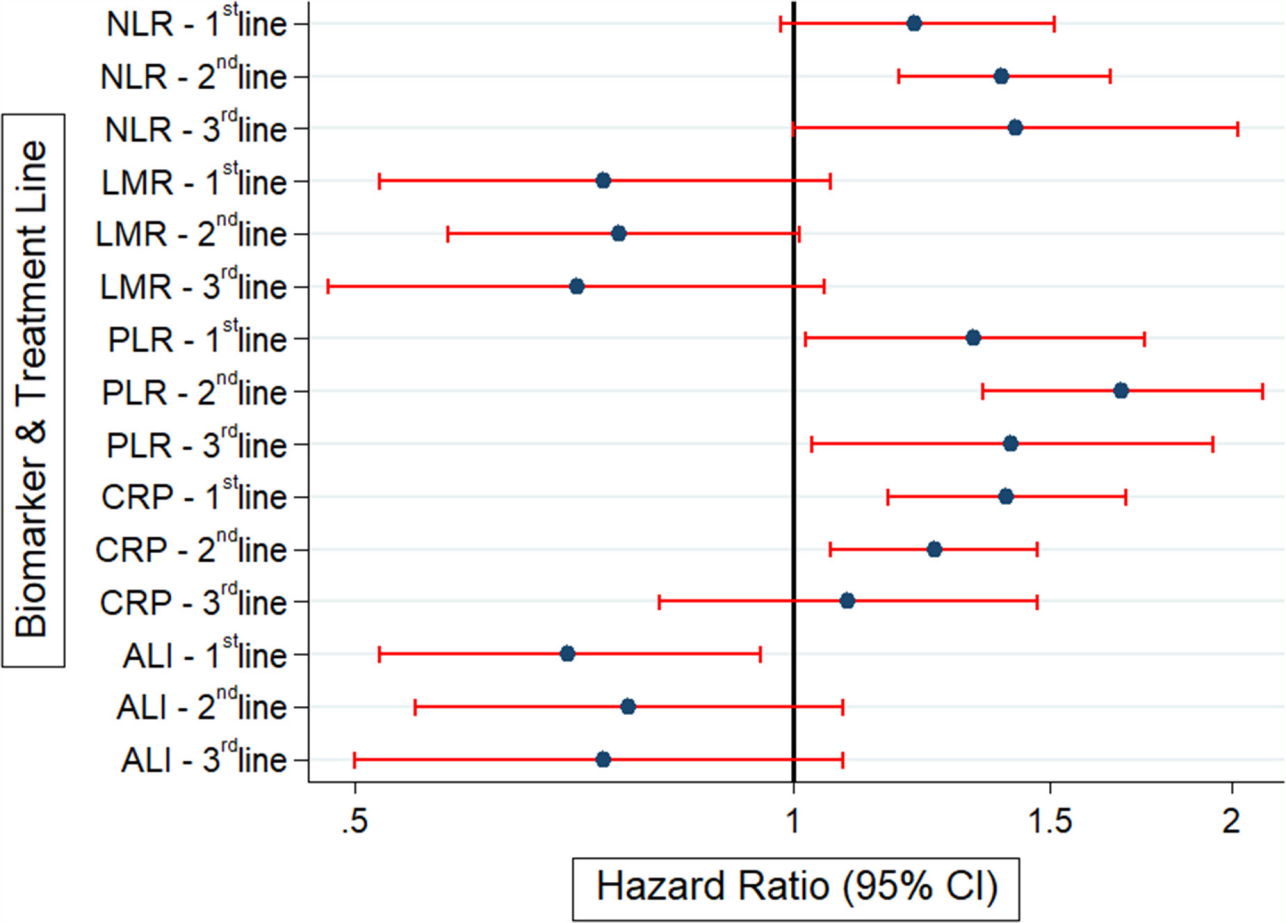
Forrest plot indicating the association between inflammatory biomarkers and the respective hazard ratio for 6 month progression free survival in first, second and third line of chemotherapy

As the BMI was not recorded for patients entering BSC, the ALI was assessable only for the first three treatment lines. In first line an elevated ALI was significantly associated with prolonged PFS, whereas in second and third line an elevated ALI was only non-significantly in favor of a better PFS experience (Figure 5A-5C). Figure 6 shows the forrest plot for associations between inflammatory biomarkers and PFS in the first three treatment lines.

### Multivariate analysis of 6-month PFS and OS across treatment lines

Besides the inflammatory biomarkers, only chemotherapy (mono- vs. polychemotherapy), but not age, sidedness or metachronous metastasis predicted for outcome (Table 3). Therefore, associations between inflammatory biomarkers and risk of progression or death across the first three treatment lines and BSC were multivariably adjusted for polychemotherapy. In this analysis, associations between inflammatory biomarkers and outcome became consistently stronger (Table 3). For instance, an elevated NLR was now strongly significantly associated with poor 6-month PFS in all three treatment lines.

## DISCUSSION

Multiple studies have shown that inflammatory biomarkers are useful prognostic tools in the first line setting of mCRC. [11] However the prognostic potential of these biomarkers in further lines of treatment and in the BSC setting of mCRC remains poorly defined. In this retrospective observational cohort study, we demonstrated that markers of systemic inflammation, namely the NLR, LMR, PLR, CRP and ALI retain their prognostic potential across multiple treatment lines in the mCRC setting, and thus appear to be useful outcome predictors beyond the first line. Furthermore, the same biomarkers emerged as predictive biomarkers for chemotherapy response. These results support the use of inflammatory biomarkers as readily available predictors of outcome and therapy response in mCRC patients across treatment lines and during treatment with BSC.

The interaction between inflammation and cancer has noticeably become the focus of cancer research in recent years. [3, 4] A strong evidence for the crucial role of inflammation in cancer development is found in colon carcinogenesis. Patients suffering from chronic bowel disease such as ulcerative colitis have a several times higher risk of developing CRC. [12] It is widely believed that reactive oxygen species build by leucocytes in chronically inflamed tissue induce DNA damage resulting in oncogenesis. In addition, cancer cells themselves release various proinflammatory cytokines to attract leucocytes which infiltrate the tumor and orchestrate the tumor microenviroment. Those inflammatory cells, in particular tumor associated macrophages produce a number of different angiogenic and growth stimulating cytokines and chemokines, which induce cancer cell proliferation and foster tumor spread. [4] In 2001, McMillan et al. could show that a high load of systemic inflammation response determined as an elevated CRP level comes along with a poor outcome in patients with advanced cancer. [13] As a consequence over recent years multiple studies have investigated the prognostic validity of various readily available inflammatory biomarkers in different cancer entities. [14–17] In metastatic colorectal cancer elevated levels of Interleukin 6, CRP and the NLR emerged as predictors of impaired disease outcome, whereas high levels of LMR seem to be associated with prolonged survival. [18–20] However all of these studies only focused on the first line setting of palliative chemotherapy. In the present study including a large cohort of mCRC patients we observed the prognostic potential of several inflammatory biomarkers over the first three therapy lines and for BSC in mCRC. First we could show that the inflammatory load measured by circulating biomarkers changes during the course of disease. The median NLR for example which seems to be a good indicator of systemic inflammation response was slightly higher in patients entering first line, than in those before second and third line, however was highly elevated in patients entering BSC. The apparent reduction of the NLR from first to second and third line has to be interpreted critically as only patients who were fit enough received further chemotherapy lines. On the contrary most patients sooner or later entered BSC, which makes it legit to compare the values of the respective biomarkers before first line and BSC. Here we observed a strong rise of systemic inflammation burden as indicated by these biomarkers. This supports the hypothesis that inflammation is a major contributor of progression and impaired survival outcome in CRC patients. [21]

Most studies use scores or cut offs determined by ROC curve analysis to analyze the association between biomarker and cancer outcome. However it is not entirely clear which threshold values are most appropriate. For instance Chua et al. who were first to investigate the prognostic and predictive value of the NLR in a large cohort of mCRC patients treated with different types of chemotherapy regimens as first line palliative treatment used a cut off NLR >5 to divide their cohort. Patients with NLR >5 had lower response rates, an increased risk of progression and a worse survival. [20] Another retrospective study by Formica et al. who observed the prognostic and predictive impact of the NLR in mCRC patients treated with FOLFIRI plus Bevacizumab as first line chemotherapy determined 3.5 as optimal NLR cut off. [22] This diversity hardens a clinician's decision which threshold should be used in clinical practice. We tried to address this issue by using two different statistical methods. First we calculated the prognostic impact of various inflammatory biomarkers by using them as continuous variables. Here we observed that high levels of NLR, PLR and CRP are not only associated with poor PFS during the first therapy line which is highly consistent with previously reported data but also in later lines of chemotherapy. High LMR and ALI seem to be favourable prognostic markers, however did not reach statistical significance. In the BSC setting an elevated CRP and low LMR emerged to be the most accurate predictors of poor OS. These data may be helpful for individual risk assessment in mCRC patients and could be used for more accurate patient stratification in clinical trials. Further, our use of continuous and Z-standardized biomarkers may enable other researchers to use our results for biomarker meta-analyses.

When using biomarkers as dichotomized variables (with empirically chosen cut-offs at the 25^th^ or 75^th^ percentile), mainly the same levels of significance could be observed for the respective biomarkers except for the NLR, where an NLR above the 3^rd^ quartile was only non-significantly associated with an impaired disease outcome compared to a NLR in the lower three quartiles during the first three therapy lines. This may be due to the loss of information and power coming along with categorization of continuous variables. [23]

The most important finding of our study was that inflammatory biomarkers do not only appear to be prognostic but also predictive tools. This concept is supported by out treatment response analysis. Here we found that particularly the NLR is a good indicator for therapy response over the first three chemotherapy lines. In detail, after adjusting for polychemotherapy 1 SD increase in NLR was associated with an 8.5% absolute lower ORR in first line, 3% lower ORR in second line and 3.1% lower ORR in third line. These results were highly significant in the first and second chemotherapy line, whereas in third line only a non-significant trend for an elevated NLR and poor therapy response could be observed. However this lack of statistical significance might be explained by the small sample size of patients entering third line chemotherapy, and should therefore not be interpreted as absence of evidence for an association. Our results are in line with previously published works on the predictive validity of the NLR in the first line setting of palliative chemotherapy. [20] However, to the best of our knowledge we were the first to investigate the predictive role of inflammatory biomarkers in further lines. These data could therefore be of clinical relevance, as they might help oncologists to identify patients who would profit from further treatment, whilst sparing patients with a low predicted benefit from side effects coming along with cytotoxic therapy treatment.

Besides the NLR right side tumor location in KRAS wildtype patients appeared to be a strong predictor of limited chemotherapy response. In detail, KRAS wildtype patients with right sided tumor location had a 25% lower ORR than those with left sided tumors in the first line of palliative chemotherapy. These results are highly consistent with a recently published retrospective analysis of the CRYSTAL and FIRE 3 trial, where right side location in RAS wildtype tumors was associated with poor treatment response and disease outcome in mCRC patients. [24].

Yet, there are some limitations that need to be discussed. First, due to its retrospective study design a selection bias in our study cohort cannot be fully excluded. Second, we did not assess potential confounding factors such as local or systemic infections, which might have affected the laboratory data collected on the inflammatory biomarkers. However, as the patients were eligible for chemotherapy in routine clinical practice, it is highly unlikely that they have suffered from severe infection at the time of biomarker measurement, which was performed within a timeframe of maximum 14 days prior to start of the respective chemotherapy line. Third, myelosuppressive chemotherapy leads to leukopenia, which may also lead to an alteration of blood based inflammatory biomarkers. This is particularly relevant when using the NLR and others in 2nd and 3rdline settings, because the patients have already been exposed to cytotoxic chemotherapy at that time. Nonetheless, the ratios between these blood cell counts remained prognostic beyond the first line setting. This suggests that the potential impact of chemotherapy on blood cell counts does not alter the prognostic potential of the investigated blood based biomarkers beyond the first line setting. Fourth, we lack an external validation cohort to verify our findings on an independent data sat. Therefore, further studies have to be performed to validate our findings. Fifth, the chemotherapy regimens administered to the patients were heterogenous. However, we aimed to test the prognostic and predictive potential of inflammatory biomarkers not only for a selected cohort receiving polychemotherapy, but for all mCRC patients treated at a Middle-European academic center. According to our opinion this might be more reflective of daily routine clinical practice.

Within the limitations of a retrospective cohort study, we conclude that our data provide strong evidence that inflammatory biomarkers are useful predictors of disease outcome and treatment response over several chemotherapy lines and best supportive care in mCRC patients and merits further validation.

## MATERIALS AND METHODS

### Study design, patient cohort, and clinical outcomes

The current study is a single-center, retrospective observational cohort study including patients with histologically-proven (metastatic) colorectal adenocarcinoma who were treated with chemo(immune-)therapy at the Clinical Division of Oncology, Medical University of Graz, Austria, between March 2010 and January 2016. These patients were drawn from our in-house colorectal cancer cohort, which includes exactly 1000 patients with UICC stage II-IV adenocarcinomas of the colon or rectum who were treated at our Department since January 2010. Of these 1000 patients, 3 were lost-to-follow-up and 612 were adjuvant patients who did not develop metastasis during a median follow-up of 2.9 years (95%CI: 2.8-3.11). Of the remaining 388 patients with metastatic disease, 130 patients did not receive any type of palliative chemotherapy (reasons: reduced performance status (n=61), declined therapy (n=13), other reasons (n=6), not known: n=50)), leaving a final analysis population of 258 patients with mCRC undergoing first-line chemo(immuno-)therapy. (Supplementary figure 1) Baseline and follow-up data were extracted from our hospital trust's electronic health record database (including all public hospitals in the province of Styria, Austria). For the main biomarker analysis, we considered 5 inflammatory biomarkers, namely the neutrophil-lymphocyte-ratio (NLR), the lymphocyte-monocyte-ratio (LMR), the platelet-lymphocyte-ratio (PLR), C-reactive protein (CRP), and the advanced lung cancer inflammation index (ALI), respectively. The ALI is defined as (body mass index ^*^ serum albumin) / NLR. We only considered laboratory data that had been collected within a timeframe of maximum 14 days prior to start of the respective chemotherapy line. In time-to-event analysis, we investigated response rates (RR) according to RECIST 1.1 criteria and rates of progression-free (PFS) during the first three lines of treatment, and overall survival (OS) after start of “best supportive care (BSC) only” treatment concept, which was defined as palliative care excluding antineoplastic therapy. Response rates were evaluated every eight weeks using CT scan. The primary endpoint was 6-month PFS during 1^st^-line, 2^nd^-line and 3^rd^-line treatment, and 6-month OS during BSC.

### Ethics statement

The study was approved by the local ethics committee (Ethikkommission der Medizinischen Universität Graz, IRB00002556) prior any patient-related activities were performed (No.25-458 ex 12/13). Written informed consent was not obtained from individual patients, because the local ethics committee specifically granted a “waiver of consent” for this retrospective database study. All investigations have been in accordance with the priniciples embodied in the declaration of Helsinki.

### Statistical analysis

All statistical analyses were performed using Stata (Windows version 14.0, Stata Corp., Houston, TX, USA). Continuous variables were summarized as medians [25^th^-75^th^ percentile], whereas categorical variables were reported as absolute counts (%). The association between response rates and the biomarkers under study were analyzed with uni- and multivariate generalized linear models from the Bernoulli family with an identity link. Median follow-up was estimated according to the method of Schemper & Smith. [25] Probabilities of progression-free and overall survival were computed with Kaplan-Meier estimators, and compared between two or more groups with log-rank tests. Uni- and multivariate modeling of PFS and OS was performed with Cox proportional hazards models. The proportionality of hazards assumption was assessed by fitting an interaction between linear follow-up time and the variables of interest. To compare the magnitude of association with PFS and OS between the different biomarkers, we Z-standardized these variables in order to render them on a common scale (mean=0, standard deviation=1).

## SUPPLEMENTARY MATERIALS FIGURES AND TABLES



## Abbreviations

NLR: neutrophil/lymphocyte ratio
LMR: lymphocyte/monocyte ratio
PLR: platelet/lymphocyte ratio
ALI: advanced lung cancer inflammation index
mCRC: metastatic colorectal cancer
PFS: progression free survival
ORR: objective response rate
SD: standard deviation
BSC: best supportive care
HR: hazard ratio
CI: confidence interval
CT: computertomography
CR: complete remission
PR: partly remission
SD: stable disease
PD: progressive disease
NE: not evaluable
DCR: disease control rate
BMI: body mass index
ROC: receiver operating characteristic

## References

[1] Siegel RL, Miller KD, Jemal A (2017). Cancer statistics, 2017. CA Cancer J Clin.

[2] Riihimäki M, Hemminki A, Sundquist J, Hemminki K (2016). Patterns of metastasis in colon and rectal cancer. Sci Rep.

[3] Mantovani A, Allavena P, Sica A, Balkwill F (2008). Cancer-related inflammation. Nature.

[4] Coussens LM, Werb Z (2002). Inflammation and cancer. Nature.

[5] Wang H, Wang L, Chi PD, Wang W, Chen XQ, Geng QR, Xia ZJ, Lu Y (2016). High level of interleukin-10 in serum predicts poor prognosis in multiple myeloma. Br J Cancer.

[6] Forrest LM, McMillan DC, McArdle CS, Angerson WJ, Dunlop DJ (2003). Evaluation of cumulative prognostic scores based on the systemic inflammatory response in patients with inoperable non-small-cell lung cancer. Br J Cancer.

[7] Shrotriya S, Walsh D, Bennani-Baiti N, Thomas S, Lorton C (2015). C-reactive protein is an important biomarker for prognosis tumor recurrence and treatment response in adult solid tumors: a systematic review. PLoS One.

[8] Proctor MJ, McMillan DC, Morrison DS, Fletcher CD, Horgan PG, Clarke SJ (2012). A derived neutrophil to lymphocyte ratio predicts survival in patients with cancer. Br J Cancer.

[9] Stotz M, Pichler M, Absenger G, Szkandera J, Arminger F, Schaberl-Moser R, Samonigg H, Stojakovic T, Gerger A (2014). The preoperative lymphocyte to monocyte ratio predicts clinical outcome in patients with stage III colon cancer. Br J Cancer.

[10] Absenger G, Szkandera J, Pichler M, Stotz M, Arminger F, Weissmueller M, Schaberl-Moser R, Samonigg H, Stojakovic T, Gerger A (2013). A derived neutrophil to lymphocyte ratio predicts clinical outcome in stage II and III colon cancer patients. Br J Cancer.

[11] Ishizuka M, Nagata H, Takagi K, Kubota K (2009). Influence of inflammation-based prognostic score on mortality of patients undergoing chemotherapy for far advanced or recurrent unresectable colorectal cancer. Ann Surg.

[12] Ekbom A, Helmick C, Zack M, Adami HO (1990). Ulcerative colitis and colorectal cancer. A population-based study. N Engl J Med.

[13] McMillan DC, Elahi MM, Sattar N, Angerson WJ, Johnstone J, McArdle CS (2001). Measurement of the systemic inflammatory response predicts cancer-specific and non-cancer survival in patients with cancer. Nutr Cancer.

[14] Richtig G, Pichler M (2017). Prediction of response in melanoma therapy by systemic inflammatory response - one size fits not all. EBioMedicine.

[15] Dalpiaz O, Luef T, Seles M, Stotz M, Stojakovic T, Pummer K, Zigeuner R, Hutterer GC, Pichler M (2017). Critical evaluation of the potential prognostic value of the pretreatment-derived neutrophil-lymphocyte ratio under consideration of C-reactive protein levels in clear cell renal cell carcinoma. Br J Cancer.

[16] Stotz M, Szkandera J, Stojakovic T, Seidel J, Samonigg H, Kornprat P, Schaberl-Moser R, Seggewies F, Hoefler G, Gerger A, Pichler M (2015). The lymphocyte to monocyte ratio in peripheral blood represents a novel prognostic marker in patients with pancreatic cancer. Clin Chem Lab Med.

[17] Stotz M, Liegl-Atzwanger B, Posch F, Mrsic E, Thalhammer M, Stojakovic T, Bezan A, Pichler M, Gerger A, Szkandera J (2016). Blood-based biomarkers are associated with disease recurrence and survival in gastrointestinal stroma tumor patients after surgical resection. PLoS One.

[18] Thomsen M, Kersten C, Sorbye H, Skovlund E, Glimelius B, Pfeiffer P, Johansen JS, Kure EH, Ikdahl T, Tveit KM, Christoffersen T, Guren TK (2016). Interleukin-6 and C-reactive protein as prognostic biomarkers in metastatic colorectal cancer. Oncotarget.

[19] Shibutani M, Maeda K, Nagahara H, Ohtani H, Sakurai K, Yamazoe S, Kimura K, Toyokawa T, Amano R, Tanaka H, Muguruma K, Hirakawa K (2015). Prognostic significance of the lymphocyte-to-monocyte ratio in patients with metastatic colorectal cancer. World J Gastroenterol.

[20] Chua W, Charles KA, Baracos VE, Clarke SJ (2011). Neutrophil/lymphocyte ratio predicts chemotherapy outcomes in patients with advanced colorectal cancer. Br J Cancer.

[21] Ghuman S, Van Hemelrijck M, Garmo H, Holmberg L, Malmstrom H, Lambe M, Hammar N, Walldius G, Jungner I, Wulaningsih W (2017). Serum inflammatory markers and colorectal cancer risk and survival. Br J Cancer.

[22] Formica V, Luccchetti J, Cunningham D, Smyth EC, Ferroni P, Nardecchia A, Tesauro M, Cereda V, Guadagni F, Roselli M (2014). Systemic inflammation, as measured by the neutrophil/lymphocyte ratio, may have differential prognostic impact before and during treatment with fluorouracil, irinotecan and bevacizumab in metastatic colorectal cancer patients. Med Oncol.

[23] Royston P, Altman DG, Sauerbrei W (2006). Dichotomizing continuous predictors in multiple regression: a bad idea. Stat Med.

[24] Tejpar S, Stintzing S, Ciardiello F, Tabernero J, Van Cutsem E, Beier F, Esser R, Lenz HJ, Heinemann V (2016). Prognostic and predictive relevance of primary tumor location in patients with RAS wild-type metastatic colorectal cancer: retrospective analyses of the CRYSTAL and FIRE-3 trials. JAMA Oncol.

[25] Schemper M, Smith TL (1996). A note on quantifying follow-up in studies of failure time. Control Clin Trials.

