# Supplementary material for: Inflammatory biomarkers in metastatic colorectal cancer: prognostic and predictive role beyond the first line setting
Source: Oncotarget. 2017 Oct 4;8(56):96048–61. doi: 10.18632/oncotarget.21647 (PMC5707080; doi:10.18632/oncotarget.21647)
Supplement: Supplementary file 1 [file oncotarget-08-96048-s001.pdf]

Inflammatory biomarkers in metastatic colorectal cancer: prognostic and predictive role beyond the first line setting

SUPPLEMENTARY MATERIALS

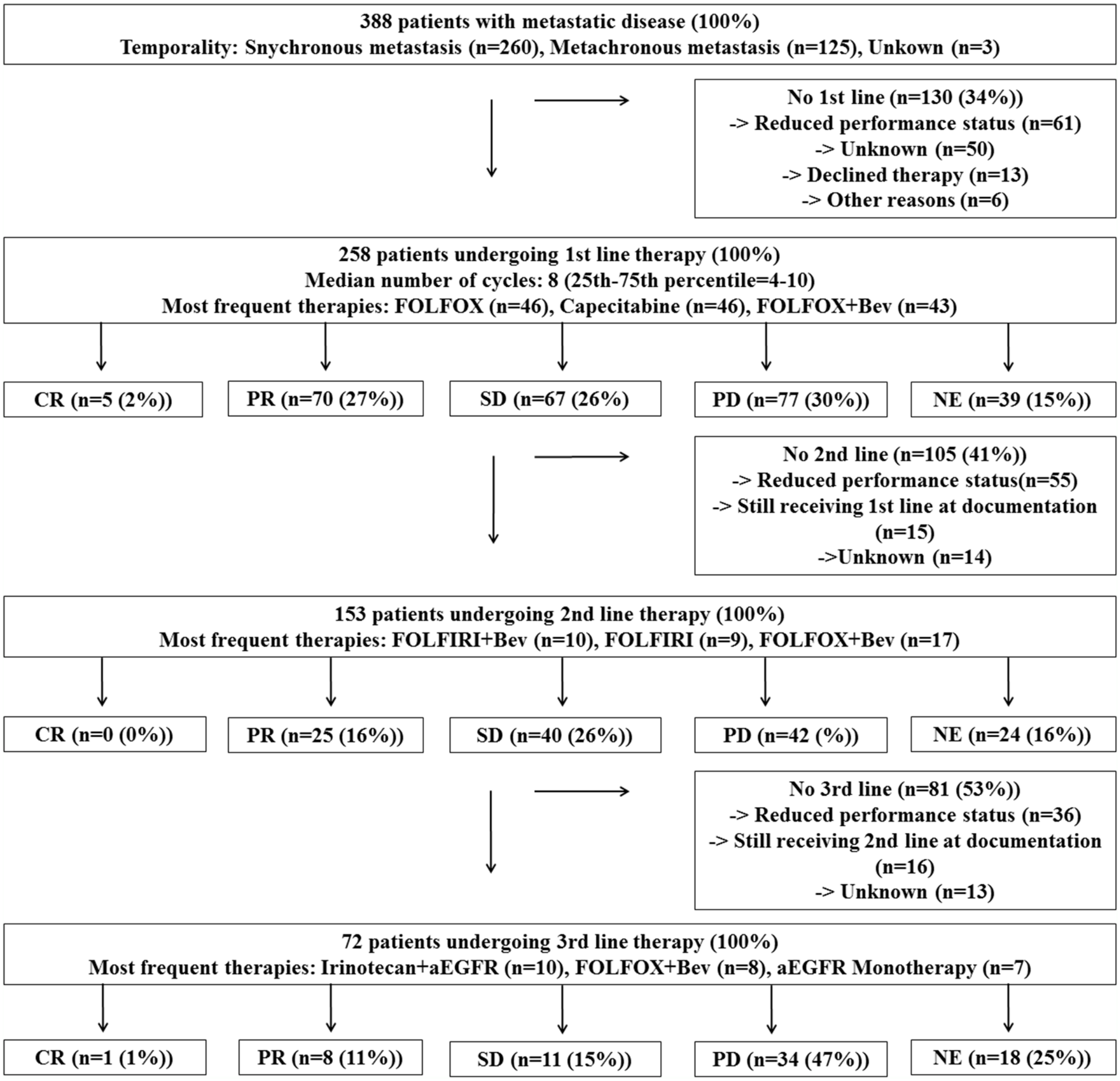

Supplementary Figure 1: Flowchart of the study population.

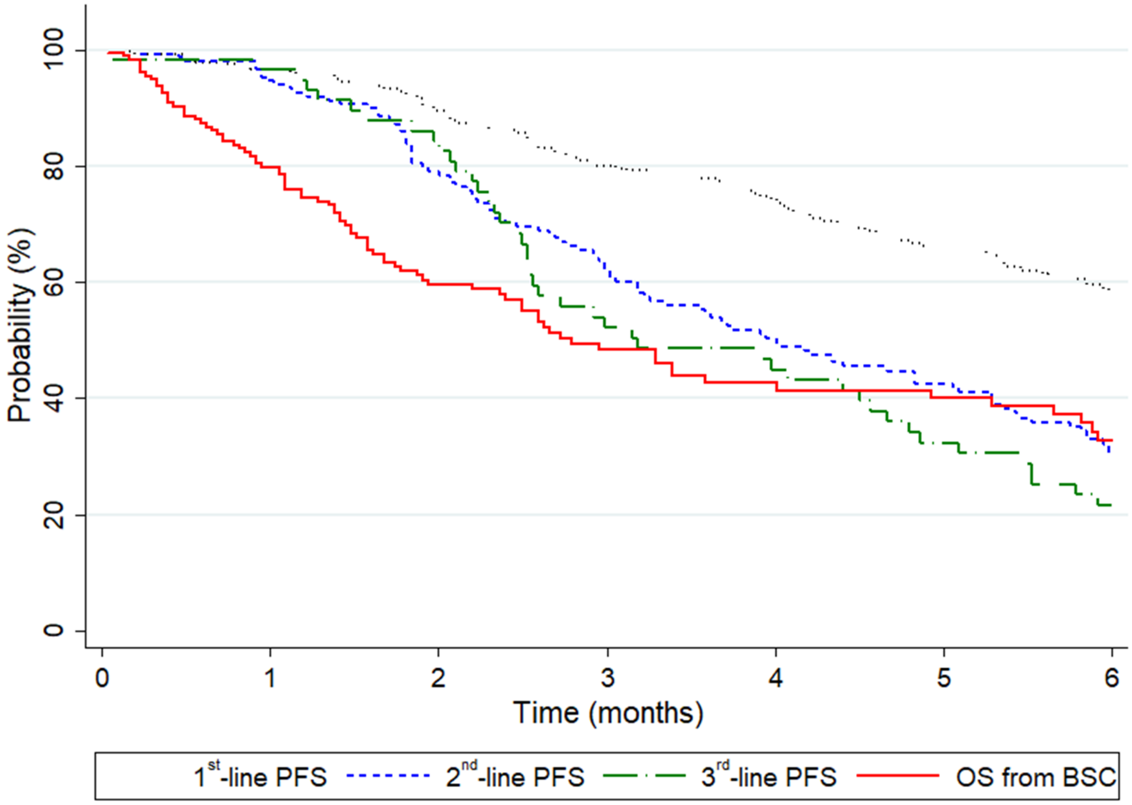

Supplementary Figure 2: Cumulative probability of progression free survival in 1st, 2nd and 3rd of palliative chemotherapy and overall survival in best supportive care.

Supplementary Table 1: Changes of inflammatory biomarkers over the first three treatment lines and BSC

	1st line (n=258)	2nd line (n=153)			3rd line (n=72)			BSC (n=183)		
	Median [25th-75th percentile]	Median [25th-75th percentile]	% relative change compared to first line	P for comparison with first line	Median [25th-75th percentile]	% relative change compared to first line	P for comparison with first line	Median [25th-75th percentile]	% relative change compared to first line	P for comparison with first line
NLR	3.9 [2.6-5.5]	3.2 [2.2-5.4]	-18 %	0.691	3.2 [1.9-5.9]	-19%	0.935	5.2 [3.1-8.5]	+32%	0.010
LMR	1.9 [1.5-2.8]	1.8 [1.2-2.8]	-6%	0.145	1.9 [1.3-3.0]	-2%	0.028	1.2 [0.8-2.0]	-36%	<0.0001
PLR	212 [147–401]	164 [123–245]	-23%	0.025	171 [115–270]	-19%	0.559	239 [155–359]	+13%	0.215
CRP	11.7 [4–34]	11 [4–34]	-9%	0.095	13.7 [5.0-48]	+17%	0.013	43 [12–96]	+268%	<0.0001
ALI*	26.9 [15.1-42.0]	30.9 [19.8-51.5]	+15%	0.345	29.5 [12.4-56.9]	+10%	0.934	N/A	N/A	N/A

Abbreviations: BSC – best supportive care, NLR – Neutrophil to lymphocyte ratio, LMR – Lymphocyte to monocyte ratio, PLR – Platelet to lymphocyte ratio, CRP – C reactive protein, ALI – Advanced lung cancer index.
